# A clinical prediction model for schizophrenia based on machine learning algorithms

**DOI:** 10.3389/fmed.2025.1726905

**Published:** 2026-01-05

**Authors:** Weifeng Jin, Shuzi Chen, Qiong Gao, Dan Li, Wei Lu, Mengxia Wang, Qing Chen, Ping Lin

**Affiliations:** Department of Medical Laboratory, Shanghai Mental Health Center, Shanghai Jiao Tong University School of Medicine, Shanghai, China

**Keywords:** schizophrenia, predictive model, machine learning, biomarkers, nomogram

## Abstract

**Objective:**

To develop an auxiliary diagnostic tool for schizophrenia based on multiple test variables using different machine learning algorithms.

**Methods:**

This retrospective study used routinely collected peripheral blood biochemical indicators, along with demographic data, to develop a diagnostic model for first-episode schizophrenia. A total of 180 patients with first-episode schizophrenia between January and August 2024, and 214 healthy controls as a population undergoing routine medical examinations during the same period. Data on age, gender, and various blood test results were collected. The dataset was divided into a training set (70%; *n* = 275) and a internal validation set (30%; *n* = 119). First, Univariate logistic regression was used to analyze significant indicators (*p* < 0.1), and feature selection was subsequently performed using the Boruta and LASSO algorithms. Machine learning models were then developed using seven machine learning algorithms, and the Area Under the Curve (AUC), Sensitivity, Specificity, Positive Predictive Value (Pos Pred Value), Negative Predictive Value (Neg Pred Value), Precision, Recall, and F1 score of each model were evaluated. Finally, we constructed an easily interpretable prediction tool based on a multiple logistic regression model. After model construction, we validated the model using an external validation set and a differential diagnosis set. A nomogram of the model outcomes was constructed, and its discrimination, calibration, and clinical decision curves were evaluated.

**Results:**

Arg, TP, ALP, HDL, UA, and LDL were ultimately identified as significant predictors through Univariate logistic regression combined with the Boruta and LASSO algorithms. The Random Forest algorithm outperformed other machine learning models, achieving an AUC of 1.00 for the training set and 0.877 for the validation set. However, due to the risk of overfitting, we ultimately selected the multivariate logistic regression model as the final model for our study and constructed nomograms.

**Conclusion:**

In this study, an auxiliary diagnostic tool for schizophrenia was established using machine learning algorithms combined with routine blood indicators. The logistic regression model demonstrated good performance and can serve as a diagnostic aid for schizophrenia.

## Introduction

Schizophrenia is a severe and disabling psychiatric disorder characterized by complex clinical manifestations and substantial functional decline. Despite major advances in neurobiological research, its diagnosis still relies predominantly on subjective symptom-based criteria outlined in the DSM-5 and ICD-11 (1), as no objective biomarkers have been incorporated into routine clinical workflows. This phenomenology-based diagnostic framework limits early detection, risk stratification, and individualized intervention.

Machine learning (ML) has emerged as a promising approach in psychiatric research, with numerous studies developing diagnostic models based on neuroimaging, electroencephalography, and multi-omics data. Although these models often demonstrate high accuracy in research settings, their clinical translation remains limited due to high acquisition costs, restricted accessibility, poor interpretability, and insufficient external validation, which together contribute to overfitting and limited generalizability (2–4). These limitations underscore the need for diagnostically useful models that balance predictive performance with feasibility and transparency.

Peripheral blood biomarkers provide an attractive alternative because they are minimally invasive, inexpensive, and widely collected in routine clinical practice. Accumulating evidence demonstrates that schizophrenia is associated with elevated inflammatory cytokines such as IL-6 and C-reactive protein (5), increased oxidative stress and impaired antioxidant defenses (6), as well as widespread abnormalities across metabolic pathways, including lipid and amino acid metabolism (7). However, because individual biomarkers lack sufficient diagnostic power, integrating multiple biochemical indicators into a composite model may yield more clinically meaningful predictive value.

The biochemical indicators selected for the present study were chosen based on biological plausibility and prior empirical evidence. Arginine, a precursor of nitric oxide, plays a central role in neuroimmune signaling, and alterations in arginine–NO metabolic pathways have been reported in schizophrenia (8). Changes in total serum protein content have been documented in proteomic studies of schizophrenia, supporting the involvement of immune and metabolic dysregulation (9). Tissue-nonspecific alkaline phosphatase (TNAP), an isoenzyme of alkaline phosphatase, contributes to endothelial function and the maintenance of blood–brain barrier (BBB) integrity, and TNAP dysregulation may be implicated in neuroinflammatory processes relevant to schizophrenia (10). Lipid abnormalities, including changes in HDL and LDL fractions, have been consistently observed among individuals with schizophrenia, suggesting altered membrane composition, lipid signaling, and metabolic dysfunction (11). Uric acid, a major endogenous antioxidant and pro-oxidant molecule, has been associated with oxidative stress imbalance in schizophrenia, supported by meta-analytic findings (12). Additionally, serum peptidomic studies further demonstrate broad alterations in circulating peptides associated with schizophrenia, reinforcing the potential utility of multi-marker blood-based models (13).

Based on these considerations, we hypothesized that a predictive model constructed from conventional serum biochemical indicators would accurately distinguish first-episode, drug-naïve patients with schizophrenia from healthy controls and would demonstrate robust generalizability in external validation. We further hypothesized that the selected biochemical indicators reflect underlying biological processes related to inflammation, oxidative stress, and metabolic dysregulation. Developing a clinically accessible and interpretable diagnostic tool has the potential to facilitate early screening and triage in psychiatric and general medical settings, particularly where specialized diagnostic resources are limited. Integration into electronic medical record systems may enable automated risk estimation and clinical decision support, although large-scale prospective validation will be necessary before routine implementation.

## Materials and methods

This retrospective study included 180 patients with first-episode schizophrenia diagnosed at the Shanghai Mental Health Center between January and August 2024, as well as 214 healthy controls who underwent physical examinations during the same period; together, these constituted the training and internal validation cohorts. Meanwhile, 164 schizophrenia patients and 192 healthy controls from the Minhang branch of the Shanghai Mental Health Center during the same period were collected as an external validation cohort. Additionally, between September 2024 and April 2025, 165 first-episode schizophrenia patients and 165 first-episode depression patients diagnosed at the Shanghai Mental Health Center were collected to serve as a validation cohort for differential diagnosis among similar disorders. The schizophrenia patients included in this study were all newly diagnosed and had not received any antipsychotic medication. Exclusion criteria for the healthy control group were as follows: individuals with comorbid or past psychiatric disorders; individuals with a history of organic brain lesions or severe traumatic brain injury; individuals with severe cardiac, hepatic, or renal insufficiency, metabolic diseases, or other serious physical illnesses; and those with abnormal results in routine laboratory tests (such as hepatic and renal function, myocardial enzymes, etc.). The results of blood tests were extracted from the information system: Albumin (ALB), Albumin-to-Globulin Ratio (A/G), Low-Density Lipoprotein (LDL), Triglyceride (TG), High-Density Lipoprotein (HDL), *γ*-Glutamyl Transferase (GGT), Alanine Transaminase (ALT), Creatinine (CREA), Alkaline Phosphatase (ALP), Blood Urea Nitrogen (BUN), Uric Acid (UA), Glucose (GLU), Total Cholesterol (TC), Total Bilirubin (T-BIL), Total Protein (TP), Arginine (Arg), Serotonin (5-HT) and Tryptophan (Trp). The sample size of this study complies with the 10EPV principle. This study was approved by the Ethics Committee of Shanghai Mental Health Center under approval number 2024 KY-190. All participants provided written informed consent. All procedures were in accordance with the ethical standards of the responsible committee on human experimentation and with the Helsinki Declaration.

## Statistical analyses

To ensure data quality, both outliers and missing values were systematically processed. First, outliers were identified using boxplots following the Tukey criterion, and observations below Q1–1.5 × IQR or above Q3 + 1.5 × IQR were removed to minimize potential bias in model training. Second, for missing data, samples with ≥20% missing variables were excluded. For samples with <20% missingness, multiple imputation with 10 imputations was performed. All imputed datasets were evaluated using AIC/BIC criteria, and the dataset with the best model fit was selected for subsequent analyses.

The extracted dataset was randomly divided into training and validation sets in a 7:3 ratio. Normally distributed variables were expressed as mean ± standard deviation (mean ± SD), while non-normally distributed variables were presented as medians (M) with interquartile ranges (IQR: P25–P75). Independent variables were initially screened using univariate logistic regression with a *p*-value threshold of <0.1. Significant variables were further selected through Lasso regression with ten-fold cross-validation (lambda 1 standard error) and the Boruta algorithm to identify the optimal combination of influencing factors. The common subset of feature variables identified by both methods was used for further analysis. Seven machine learning algorithms were developed using R, including Decision Trees, Random Forests, XGBoost, K-Nearest Neighbors, LightGBM, Naive Bayes, and logistic regression. Hyperparameter tuning was performed within the training set using repeated 10-fold cross-validation (repeated 10-fold CV × 5). A grid search over predefined hyperparameter grids was conducted, and the combination yielding the highest mean AUC across all folds and repetitions was selected as the optimal configuration. The main tuning parameters for each algorithm were as follows: Decision Trees cp, maxdepth, Random Forests: mtry, ntree, XGBoost: eta, max_depth, gamma, subsample, colsample_bytree, nrounds, KNN: number of neighbors (k), distance metric, kernel, LightGBM: num_leaves, max_depth, learning_rate, feature_fraction, bagging_fraction, Naive Bayes: usekernel, laplace, Logistic regression: no hyperparameter tuning required. All tuning procedures were conducted exclusively within the training set, while the validation and external datasets were used solely to evaluate the final model performance. The comparisons of AUCs between different models were performed using DeLong’s test. Given that logistic regression is interpretable and relatively simple, we constructed a nomogram based on the multivariable logistic regression model and evaluated its discrimination, calibration, and clinical applicability. All statistical analyses and machine learning models were implemented in R (version 4.2.1) using the following packages: Boruta, glmnet, rpart, randomForest, xgboost, kknn, lightgbm, glm, rms, and ROCR.

## Result

### Baseline analysis

The data from the included studies were randomly divided into training and validation sets in a 7:3 ratio. The baseline features of the training set, internal validation set, external validation set, and differential diagnosis set are shown in Table 1. All indicators were analyzed for differences with the training set.

**Table 1:** Analysis of baseline data for training and validation sets.

Parameters	Train *N* = 275	Internal validation set *N* = 119	External validation set *N* = 356	Differential diagnosis set *N* = 449
Gender				
Male	104 (37.82%)	46 (38.66%)	141 (39.61%)	158 (35.19%)
Female	171 (62.18%)	73 (61.34%)	215 (60.39%)	291 (64.81%)
Age	38.70 ± 10.79	38.91 ± 10.50	37.90 ± 8.55	38.82 ± 17.08
ALB (g/L)	43.80 [41.60; 45.95]	44.40 [42.30; 46.40]	44.00 [42.18; 45.62]	42.50 [40.30; 45.07] ***
A/G	1.57 [1.38; 1.91]	1.55 [1.42; 1.96]	1.55 [1.42; 2.01]	1.64 [1.44; 2.02]
LDL (mmol/L)	2.47 [1.99; 2.94]	2.49 [2.05; 2.89]	2.46 [2.13; 2.87]	2.48 [2.07; 3.00]
TG (mmol/L)	1.12 [0.81; 1.60]	1.10 [0.82; 1.74]	1.23 [0.91; 1.67] *	1.21 [0.87; 1.75]
HDL (mmol/L)	1.11 [0.92; 1.33]	1.15 [0.94; 1.31]	1.12 [0.97; 1.32]	1.13 [0.94; 1.35]
GGT (IU/L)	20.00 [15.00; 32.50]	22.00 [14.50; 32.50]	21.00 [15.00; 31.00]	22.00 [16.00; 33.00]
ALT (IU/L)	19.00 [13.00; 28.50]	20.00 [12.50; 28.00]	19.00 [14.00; 27.00]	19.00 [13.00; 27.00]
CREA (umol/L)	65.00 [56.00; 76.50]	65.00 [57.50; 75.00]	65.00 [57.00; 74.00]	64.00 [55.00; 74.00]
ALP (IU/L)	64.00 [53.50; 75.00]	65.00 [53.00; 76.00]	65.00 [55.75; 72.00]	67.00 [56.00; 79.00] *
BUN (mmol/L)	4.35 [3.62; 5.36]	4.31 [3.63; 5.07]	4.36 [3.73; 5.13]	4.22 [3.61; 5.25]
UA (umol/L)	359.00 [295.00; 429.50]	367.00 [297.00; 444.00]	356.00 [297.00; 428.25]	327.00 [267.00; 417.00] **
GLU (mmol/L)	5.01 [4.68; 5.50]	5.14 [4.70; 5.62]	5.11 [4.76; 5.47]	5.25 [4.63; 6.04] *
TC (mmol/L)	4.75 [4.06; 5.44]	4.73 [4.16; 5.31]	4.78 [4.30; 5.28]	4.68 [4.08; 5.39]
T-BIL (umol/L)	11.60 [9.23; 15.14]	11.24 [9.16; 14.82]	11.97 [9.64; 14.75]	11.21 [9.24; 14.63]
TP (g/L)	73.20 [69.70; 76.45]	73.60 [71.00; 77.20]	73.90 [70.88; 76.20]	71.90 [68.40; 75.50] *
Trp (μg/mL)	8.44 [7.26; 10.32]	8.64 [7.36; 10.17]	8.43 [7.40; 9.99]	9.22 [7.96; 10.96] ***
5HT (ng/mL)	138.68 [90.65; 195.62]	131.32 [90.38; 199.45]	132.46 [95.50; 195.44]	47.81 [8.54; 119.40] ***
Arg (μg/mL)	17.73 [14.25; 21.23]	17.32 [14.59; 20.51]	8.27 [6.66; 10.60]	16.81 [14.32; 19.88] **

Comparing each validation set to the training set * *p*-value < 0.05; ** *p*-value < 0.01; *** *p*-value < 0.001.

### Univariate logistic regression

Univariate logistic regression analysis was performed on all blood indicators within the training set, retaining variables with *p*-values <0.1 for further analysis.

The statistically significant indicators included Arg, TP, ALP, HDL, ALB, TC, UA, LDL, and TG, presented in Table 2.

**Table 2:** Univariate logistic regression analysis in the training set.

Characteristics	*B*	SE	OR (95% CI)	*P*
Arg (μg/mL)	−0.111	0.02486	0.895 (0.85–0.937)	**0**
5HT (ng/mL)	0	0.00116	1 (0.998–1.002)	0.985
Trp (μg/mL)	−0.045	0.04799	0.956 (0.869–1.05)	0.35
TP (g/L)	−0.215	0.0327	0.807 (0.754–0.858)	**0**
T-BIL (umol/L)	−0.033	0.02234	0.967 (0.924–1.009)	0.134
TC (mmol/L)	−0.357	0.12556	0.7 (0.544–0.892)	**0.005**
GLU (mmol/L)	0.139	0.08609	1.149 (0.981–1.383)	0.107
UA (umol/L)	0.003	0.00122	1.003 (1.001–1.006)	**0.01**
BUN (mmol/L)	0.046	0.08389	1.047 (0.888–1.236)	0.583
ALP (IU/L)	0.03	0.00753	1.03 (1.015–1.046)	**0**
CREA (umol/L)	0.005	0.00831	1.005 (0.989–1.022)	0.51
ALT (IU/L)	0.004	0.00597	1.004 (0.992–1.016)	0.493
GGT (IU/L)	0.006	0.00563	1.006 (0.995–1.018)	0.279
HDL (mmol/L)	−1.76	0.42494	0.172 (0.073–0.386)	**0**
TG (mmol/L)	0.259	0.13593	1.296 (1.004–1.721)	**0.056**
LDL (mmol/L)	−0.403	0.17855	0.668 (0.468–0.944)	**0.024**
A/G	0.449	0.61054	1.566 (1.12–5.268)	0.462
ALB (g/L)	−0.187	0.04155	0.829 (0.762–0.897)	**0**

Bold values indicate *p*-values < 0.1.

### Lasso regression and Boruta algorithm for feature screening

**Figure 1:**
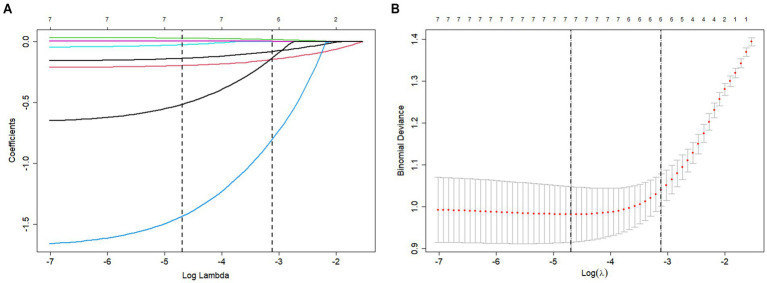
**(A)** Lasso log lambda. **(B)** Lasso regression with cross-validation.

**Figure 2:**
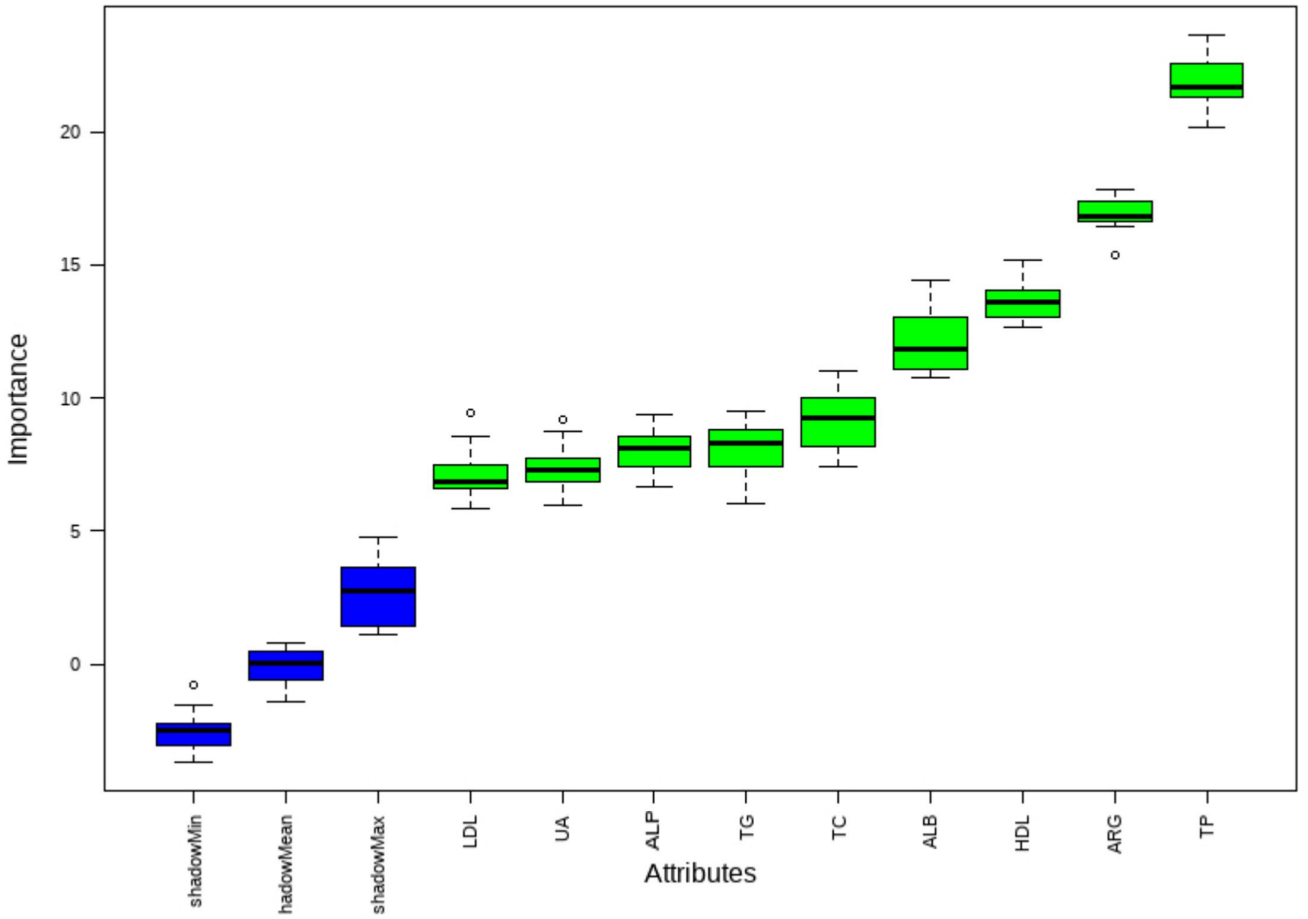
Boruta screening.

Lasso regression was performed to further analyze the statistically significant factors identified above. The non-zero coefficients at Lambda.1se, including Arg, TP, ALP, HDL, UA, and LDL, are presented in Figure 1. Boruta screening with 100 iterations was then applied to further analyze these factors. The variables identified by Boruta, including Arg, TP, ALP, HDL, UA, LDL, ALB, TC, and TG, are shown in Figure 2.

### Machine learning model evaluation

**Figure 3:**
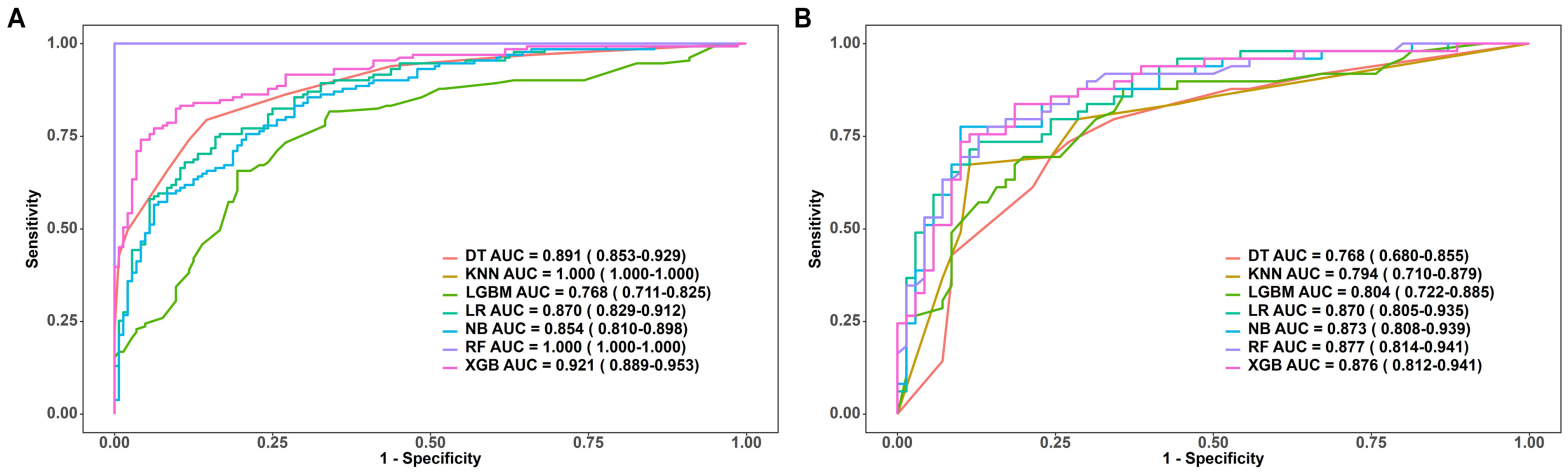
**(A)** AUCs of 7 machine learning models on the training set. **(B)** AUCs of 7 machine learning models on the internal validation set.

After screening by LASSO regression and Boruta, we applied seven machine learning algorithms to construct and evaluate predictive models: Decision Trees (DT), Random Forests (RF), Extreme Gradient Boosting (XGBoost), K-Nearest Neighbors (KNN), Light Gradient Boosting Machines (LightGBM), Naive Bayes (NB), and Logistic Regression (LR). A comprehensive model incorporating all selected variables was constructed. The performance of the seven models was evaluated using metrics such as AUC, sensitivity, specificity, PPV, NPV, precision-recall, and F1-score, as summarized in Table 3. The AUC values of the models are also visualized in Figure 3.

**Table 3:** Predictive performance comparison of the seven types of machine learning algorithms.

Model	Different classifications	AUC	Sensitivity	Specificity	Pos pred value	Neg pred value	Precision	Recall	F1
LR	dev	0.87	0.756	0.833	0.805	0.789	0.805	0.756	0.78
DT	dev	0.891	0.832	0.82	0.794	0.854	0.794	0.832	0.812
RF	dev	1	1	1	1	1	1	1	1
XGB	dev	0.921	0.832	0.896	0.879	0.854	0.879	0.832	0.855
KNN	dev	1	1	1	1	1	1	1	1
LGBM	dev	0.768	0.817	0.66	0.686	0.798	0.686	0.817	0.746
NBM	dev	0.854	0.855	0.694	0.718	0.84	0.718	0.855	0.78
LR	vad	0.87	0.796	0.743	0.684	0.839	0.684	0.796	0.736
DT	vad	0.768	0.655	0.797	0.735	0.729	0.735	0.655	0.692
RF	vad	0.877	0.796	0.8	0.736	0.848	0.736	0.796	0.765
XGB	vad	0.876	0.796	0.814	0.75	0.851	0.75	0.796	0.772
KNN	vad	0.794	0.694	0.757	0.667	0.779	0.667	0.694	0.68
LGBM	vad	0.804	0.857	0.643	0.627	0.865	0.627	0.857	0.724
NBM	vad	0.873	0.878	0.643	0.632	0.882	0.632	0.878	0.735

**Figure 4:**
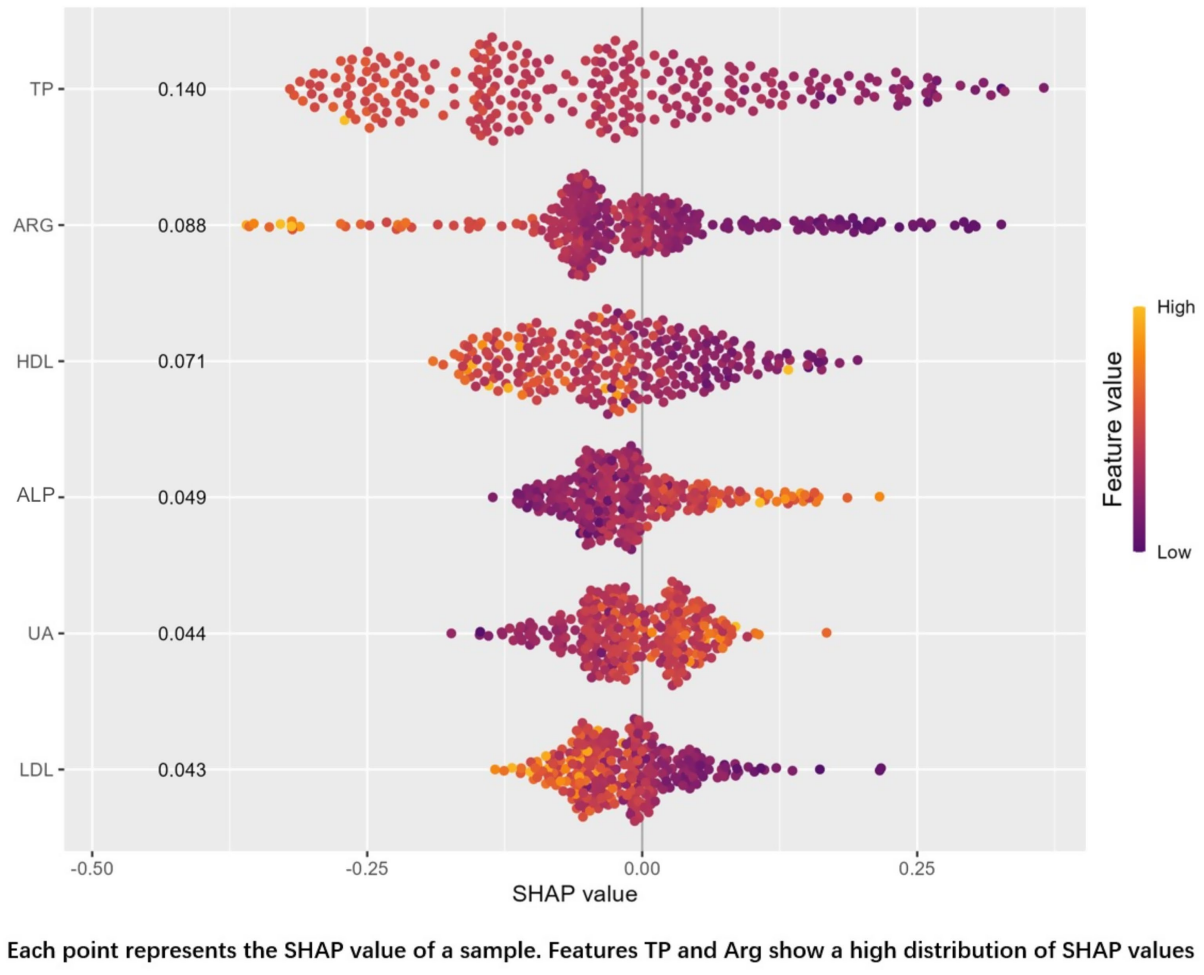
SHAP diagram.

Among the seven models, the Random Forest (RF) model achieved the highest AUC on the validation set. To further understand its predictions, we performed a SHAP (SHapley Additive exPlanations) analysis, as shown in Figure 4. Despite the superior AUC of the RF model (0.877) on the validation set, concerns about potential overfitting prompted further comparison with the Logistic Regression (LR) model, which achieved a similar AUC (0.870). The difference between the two models’ AUCs was not statistically significant (*p* = 0.877). Ultimately, we selected the Logistic Regression model as the optimal predictive model due to its simplicity and interpretability.

### Multivariate logistic regression and predictive model development

A multifactor logistic regression prediction model was constructed, with disease diagnosis as the dependent variable, and the results are shown in Table 4. The final multifactor logistic regression formula was derived: *LogitP* = 20.16–0.67*LDL-1.66*HDL + 0.031*ALP + 0.004*UA-0.236*TP-0.16*Arg. The variance inflation factors (VIF) for all variables included in the model were less than 10, indicating the absence of multicollinearity.

**Table 4:** Multivariate logistic regression analysis in the training set.

Characteristics	B	SE	OR (95%CI)	*P*
LDL	−0.666	0.2392	0.513 (0.316–0.812)	0.005
HDL	−1.661	0.5454	0.189 (0.063–0.539)	0.002
ALP	0.031	0.0093	1.031 (1.013–1.051)	0.001
UA	0.004	0.00163	1.003 (1.001–1.007)	0.015
TP	−0.236	0.03929	0.789 (0.727–0.849)	0
Arg	−0.16	0.03027	0.851 (0.799–0.901)	0

### Predictive model performance evaluation

**Figure 5:**
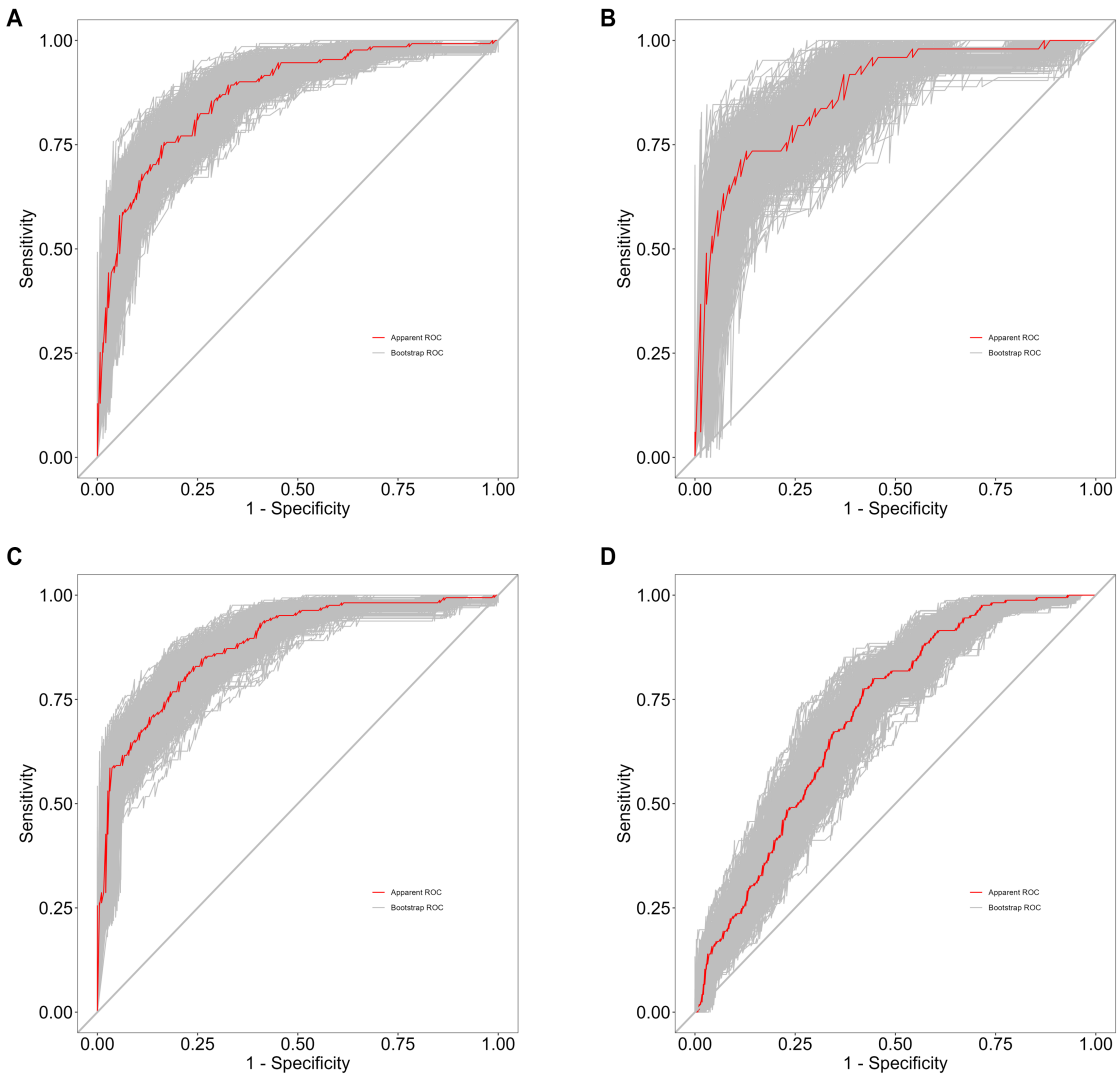
**(A)** Training set of 500 bootstrap ROC curves. **(B)** Internal validation set of 500 bootstrap ROC curves. **(C)** External validation set of 500 bootstrap ROC curves. **(D)** Differential diagnosis set of 500 bootstrap ROC curves.

The performance of the predictive model was evaluated based on its discrimination and calibration abilities. After 500 bootstrap samples, the areas under the ROC curve (AUC) for the model in the training set, internal validation set, external validation set, and differential diagnosis set were 0.8763 (95% CI: 0.8342–0.9183), 0.9021 (95% CI: 0.8478–0.9564), 0.8753 (95% CI: 0.8404–0.9102), and 0.7071 (95% CI: 0.66–0.7542), respectively. These results indicate that the model performed well in distinguishing schizophrenia patients from healthy controls, but further improvement is needed for differentiating between schizophrenia and depressive disorder, as shown in Figure 5.

**Figure 6:**
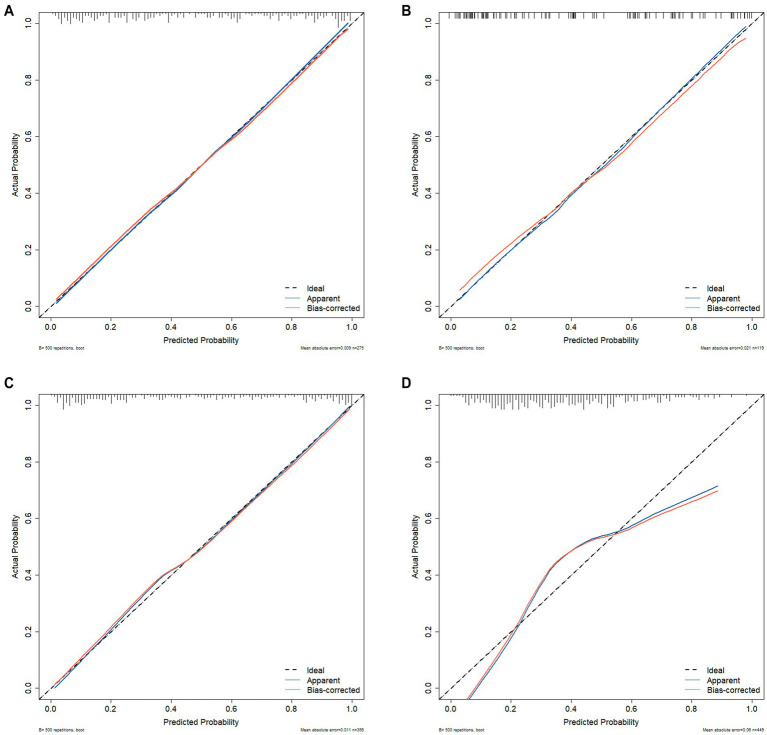
**(A)** Training set of 500 bootstrap calibration curves. **(B)** Internal validation set of 500 bootstrap calibration curves. **(C)** External validation set of 500 bootstrap calibration curves. **(D)** Differential diagnosis set of 500 bootstrap calibration curves.

We also assessed the predictive performance of the clinical prediction model. The Brier scores for the training set (0.146), internal validation set (0.150), external validation set (0.140), and differential diagnosis set (0.248) indicate that the model had low error and high accuracy in probability predictions. The calibration curves based on 500 bootstrap samples are shown in Figure 6. In addition, the Hosmer–Lemeshow calibration test *p*-values for the training set, internal validation set, external validation set, and differential diagnosis set were 0.5874, 0.162, 0.1494, and <0.05, respectively. This suggests that there was no significant difference between predicted probabilities and actual outcomes when distinguishing the schizophrenia group from healthy controls, but calibration performance was poorer when distinguishing between schizophrenia and depressive disorder.

The performance parameters of the logistic regression model in the external validation cohort and the diagnostic cohort are detailed in Table 5.

**Table 5:** Performance metrics of logistic regression models in the external validation cohort and diagnostic cohort.

Model	AUC	Sensitivity	Specificity	Pos pred value	Neg pred value	Precision	Recall	F1
External validation cohort	0.8753	0.713	0.854	0.807	0.777	0.807	0.713	0.757
Diagnostic cohort	0.7071	0.752	0.588	0.515	0.803	0.515	0.752	0.611

### Nomogram construction and assessment of clinical efficacy

**Figure 7:**
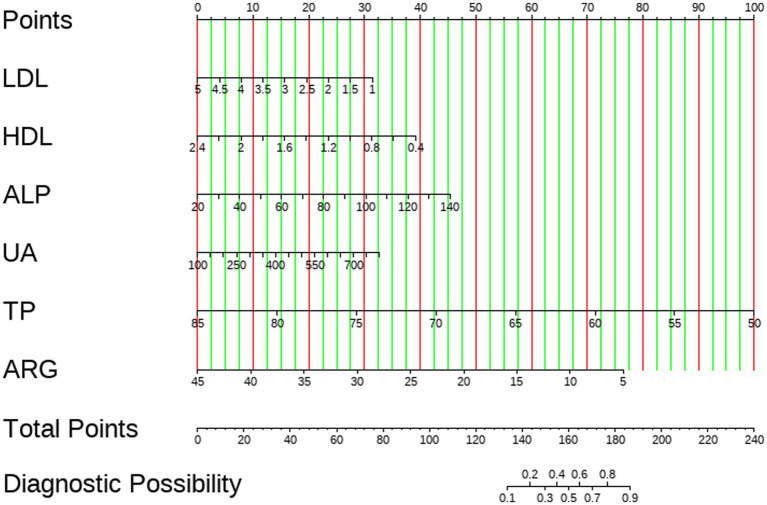
Nomogram for predicting schizophrenia.

**Figure 8:**
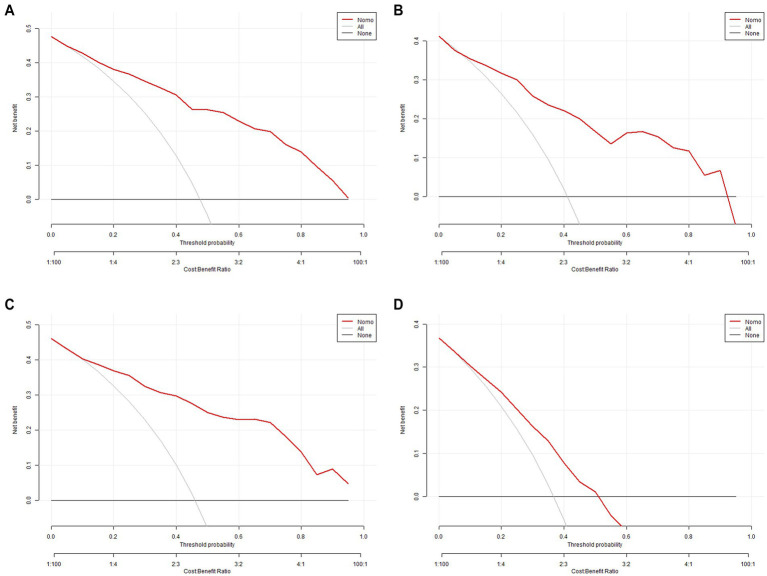
**(A)** DCA for the training set. **(B)** DCA for the internal validation. **(C)** DCA for the external validation. **(D)** DCA for the differential diagnosis set.

A nomogram for predicting schizophrenia was developed by incorporating six significant predictors identified through multivariate analysis. The visual representation of the predictive model is presented as a nomogram in Figure 7. The clinical utility of the model was evaluated using decision curve analysis. The results demonstrated that the range of threshold probabilities for achieving maximum net benefit was 0.05 to 100% in the training set, 0.07 to 93% in the internal validation set, 0.05 to 100% in the external validation set, and 0.08 to 52% in the differential diagnosis set, as shown in Figure 8.

## Discussion

In this study, an auxiliary diagnostic tool for schizophrenia was developed based on the results of seven machine learning approaches. Among these approaches, Random Forest demonstrated the best performance, with an AUC of 1.00 in the training set and 0.877 in the validation set, as visualized using SHAP analysis. The variable importance rankings of the random forest model were: TP, Arg, HDL, ALP, UA, LDL, while those of the logistic regression model based on odds ratios were: HDL, LDL, TP, Arg, ALP, UA. This difference may be primarily attributed to the fact that logistic regression is a linear model, where variable importance is determined by coefficient magnitude, reflecting the strength of linear relationships with the target variable, while random forest is a non-linear model, where variable importance is based on decision tree split effectiveness, considering the overall predictive power of the variables. Logistic regression was ultimately selected as the final model to mitigate the risk of overfitting.

In Góngora Alonso et al.’s study, the Random Forest algorithm achieved an AUC of 0.796 in predicting inpatient schizophrenia patients, demonstrating strong discriminatory ability (14). Similarly, Yupeng He et al.’s SZ classifier achieved an AUC of 0.86 in internal validation. However, in external validation, especially when differentiating patients with bipolar disorder and major depression, the misclassification rate exceeded 50%, highlighting challenges in distinguishing schizophrenia from other psychiatric disorders (15). Additionally, Carmen Soria Bretones et al. analyzed EEG recordings using a deep learning-based system, achieving an AUC of approximately 0.93 and demonstrating very high differentiation accuracy (16). When the model was used to distinguish between similar diseases, it demonstrated moderate discriminatory ability (AUC 0.7071). The model maintained relatively robust sensitivity (0.752) and negative predictive value (0.803) when distinguishing between schizophrenia and depression, indicating that it has some value in identifying true schizophrenia cases and providing reliable negative predictive results, potentially aiding in the preliminary exclusion of schizophrenia in complex cases. However, the model’s performance in the diagnostic set was somewhat lower than that in the training set, particularly in terms of specificity (0.588) and positive predictive value (0.515), which suggest room for improvement. This indicates that the model faces challenges in distinguishing between similar diseases and that its generalization performance needs to be optimized. The F1 score (0.611) also reflects the model’s potential for improvement in this complex differential diagnosis task. This result indicates that the model is currently suitable for distinguishing between healthy individuals and schizophrenia, suggesting that future research could further explore incorporating more disease-specific features or optimizing the model strategy to enhance its accuracy and robustness in distinguishing such mentally disorders with similar phenotypes.

In contrast to these studies, our model utilizes conventional blood indicators, achieving an AUC of 0.87 in both the training and validation sets. This performance is comparable to existing studies in terms of discriminatory accuracy, while offering significant advantages in data accessibility and model simplicity. By relying on non-invasive and easily collected blood data, our study simplifies clinical application while maintaining a high level of discrimination, which is critical for the early identification and intervention of schizophrenia.

The logistic regression results in this study showed that ALP and UA were positively correlated with schizophrenia, while LDL, HDL, TP, and Arg were negatively correlated. Serum uric acid (UA) levels were found to be significantly higher in patients with schizophrenia compared to normal controls, consistent with the findings of Canlı and Demir. Their study reported elevated UA levels in patients with schizophrenia, schizoaffective disorder, and bipolar disorder compared to healthy controls, which may be attributed to oxidative stress and alterations in the purinergic system (17). Ma et al. proposed that uric acid, a natural antioxidant, is influenced by various factors in patients with schizophrenia, including oxidative stress, cellular damage, drug use, metabolic disorders, nutritional status, lifestyle factors, smoking, and ethnic background (18).

The specific role of alkaline phosphatase (ALP) in the blood–brain barrier (BBB) is mainly reflected in its regulation of BBB integrity and function. Tissue-nonspecific alkaline phosphatase (TNAP) is highly expressed in brain microvascular endothelial cells (BMECs) and participates in maintaining the integrity of the BBB through the Rho-associated protein kinase (ROCK) pathway. Moreover, TNAP activity is closely related to the barrier function of the BBB, and its inhibition can lead to BBB dysfunction and exacerbation of inflammatory responses (19). In inflammatory diseases such as sepsis, TNAP activity in key brain regions is decreased, resulting in impaired BBB function, promoting leukocyte infiltration into the brain parenchyma and the release of pro-inflammatory cytokines, thereby inducing neuroinflammation and long-term cognitive dysfunction (20). Therefore, TNAP plays an important role in maintaining BBB integrity and regulating neuroinflammation. Changes in ALP levels, potentially linked to immune responses, may reflect the immunopathology of schizophrenia (21).

Our study observed that patients with schizophrenia had lower levels of HDL and LDL than healthy controls, a finding that is in line with Tian et al. who found that patients with schizophrenia in the acute phase and who were not on medication had lower lipid metabolic parameters (22). In addition, a review by Goh et al. emphasized the possible role of dysfunction of the oxytocin system in the association of schizophrenia with the metabolic syndrome, which may affect lipid metabolism (23). Our study also found reduced serum total protein in patients with schizophrenia this is in line with the findings of Yin et al. (24).

Arginine is a conditionally essential amino acid that plays a key role in central nervous system function and immune regulation. Beyond its basic metabolic roles, arginine is the substrate for nitric oxide synthase (NOS), which produces nitric oxide (NO)—a critical neuromodulator involved in glutamatergic transmission. Dysregulation of the arginine–NO pathway has been shown to influence NMDA receptor function: insufficient NO signaling may reduce NMDA receptor activation and impair synaptic plasticity, whereas excessive NO production can trigger oxidative and nitrosative stress, contributing to neuronal dysfunction (25). Moreover, shunting of arginine metabolism toward the arginase pathway can decrease NO bioavailability and increase polyamine synthesis, mechanisms associated with neuroinflammation and excitatory–inhibitory imbalance (26). The bidirectional interaction between NO and NMDA receptor activity—where NMDA receptor–mediated Ca^2+^ influx activates NOS, and NO subsequently modulates glutamate release—provides a plausible mechanistic link between peripheral metabolic disturbances and central neurotransmission abnormalities in schizophrenia (27, 28). Our findings show that serum arginine levels are reduced in patients with schizophrenia. This is consistent with evidence from postmortem brain analyses demonstrating altered arginine metabolites in schizophrenia (29) and serum metabolomic studies reporting decreased arginine concentrations in chronic schizophrenia (30). Together, these results suggest that disruptions in arginine metabolism may contribute to NO signaling abnormalities and NMDA receptor–related dysfunction, supporting a potential role for the arginine–NO pathway in the pathophysiology of schizophrenia.

In this study, we analyzed independent risk factors among blood indicators for schizophrenia and developed a visual nomogram accordingly. The model demonstrated strong predictive performance in terms of discrimination, calibration, and clinical efficacy. Notwithstanding its findings, this study has several limitations. First, the inclusion of participants from a single institution and exclusively of Chinese descent may introduce homogeneity in genetic background, lifestyle patterns, and clinical characteristics. Consequently, the generalizability of our model may be limited, and its performance might vary across diverse geographic regions, healthcare systems, or ethnic groups. Furthermore, the retrospective nature of this study design carries an inherent risk of selection bias. To minimize this bias, we adopted a consecutive enrollment strategy, including all eligible subjects during the study period. Nevertheless, the inherent limitations of the retrospective design may still affect the interpretation of our results. Another limitation of this study is that although we used the Minhang Campus as an external validation set, strictly speaking, the Minhang Campus and the Shanghai Mental Health Center belong to the same medical system. In the future, our research team will select completely independent external data to validate the model. Additionally, due to the retrospective nature of this study, certain parameters that may influence metabolism (such as smoking status and BMI) were unavailable. Although our model identified low-density lipoprotein (LDL), uric acid (UA), high-density lipoprotein (HDL), and other biomarkers as predictive factors, we acknowledge that the absence of smoking and body mass index (BMI) data may introduce confounding factors. Epidemiological studies have shown that smoking can increase uric acid levels by approximately 20% (31), HDL-C levels are significantly lower in smokers compared to non-smokers (32), and LDL-C levels are on average 11.0% higher (33); obesity (BMI > 30) can lead to an increase in LDL-C levels of 15–30% (34). Future work should therefore include multicenter, prospective validation to enhance robustness. Additionally, incorporating lifestyle-related variables (e.g., diet, physical activity, smoking, sleep) may provide a more comprehensive representation of individual health status. Integrating routine blood indicators with imaging features or digital phenotypes (such as wearable device data or behavioral markers) could further improve the predictive accuracy and clinical utility of future models.

In summary, the schizophrenia prediction model based on Arg, TP, ALP, HDL, UA, and LDL provides an objective and reliable tool for clinical auxiliary diagnosis.

## Data Availability

The raw data supporting the conclusions of this article will be made available by the authors, without undue reservation.
